# Place of sanctuary: an appreciative inquiry approach to discovering how communities support breastfeeding and parenting

**DOI:** 10.1186/s13006-019-0219-8

**Published:** 2019-06-11

**Authors:** Virginia Schmied, Elaine Burns, Athena Sheehan

**Affiliations:** School of Nursing and Midwifery, Western Sydney University, Locked Bag 1797, Parramatta, NSW 2751 Australia

**Keywords:** Breastfeeding, Public spaces, Place, Community, Appreciative inquiry, Green spaces, Retail outlets, Shopping centres

## Abstract

**Background:**

Significant efforts by governments at a global and national level have not resulted in a significant increase in the duration of breastfeeding to six months. The views of family and social networks, and community attitudes particularly around breastfeeding in public, influence infant feeding decisions. Yet many interventions designed to increase breastfeeding focus on the individual woman and have not been developed from the ‘ground up’ in consultation with women and communities. This study aimed to identify the key components of Mother Infant Caring Communities that promote and support breastfeeding and early parenting.

**Methods:**

Appreciative Inquiry was used to facilitate a ‘Community Conversation’ workshop in two local councils in Australia. Thirty-five participants attended the community conversation workshops including new parents, grandparents, children’s services, local government, and representatives from maternity and child health services. In addition, one focus group discussion was conducted with six retail business owners or managers. Qualitative content analysis was used to analyse data. This paper presents the findings of the first phase (the Discovery phase) of the study.

**Results:**

Four major themes emerged: “PLACE – A community for everyone”; “A PLACE for children and families”; “Sometimes a PLACE to breastfeed” and “The parent room: a hidden and unsafe PLACE to breastfeed”. Participants described the characteristics of communities that provided a sanctuary and fostered well-being for parents and infants including, open green spaces, safe playgrounds, walking tracks and community hubs. Shopping centres were described as having the potential to be the ‘village’. Community-based services to support breastfeeding and parenting were highly valued. Yet in both sites, participants stated that breastfeeding was rarely observed in public and bottle feeding was more evident.

**Conclusion:**

Breastfeeding and parenting are embedded in the places where women and families live. Community spaces including shopping centres, should be designed to include infants and young children and offer appropriate facilities such as safe and clean parenting rooms. Health services must work with local government, businesses, and diverse community members to identify what parents’ value about their community and design and implement innovative local strategies to support breastfeeding.

## Background

Parents face many decisions concerning parenting practices. The first three years of the infant’s life, in particular, are crucial in laying the foundation for health and well-being in later life [1]. Breastfeeding is known to have important short- and long-term benefits for children and for mothers [2, 3]. In addition to health benefits, breastfeeding is also viewed as the most ecologically sustainable way to feed infants and provides substantial cost savings to families, the health care system, employers and government [4]. In Australia*,* the National Infant Feeding Survey 2010 [5] reported breastfeeding was initiated for 96% of children aged 0–2 years and around 69% of infants were still receiving some breast milk at four months of age, but only 39% were exclusively breastfed to three months, and only 15% were exclusively breastfed to five completed months of age. More recently, the New South Wales Mothers and Babies 2016 report demonstrated the percentage of babies fully breastfed at the time of discharge from hospital had decreased from 82.1 to 74.9% between 2012 and 2016 [6].

Public health policies, while promoting breastfeeding, have tended to focus on the immunological and biological benefits for infant health and on testing interventions to increase breastfeeding initiation and duration with less emphasis on understanding the socio-cultural and community influences on infant feeding decisions. A recent review undertaken for the Australian Breastfeeding Strategy identified that the majority of studies testing interventions to promote and support breastfeeding, occur within the health system and less focus is on interventions delivered in the home and family, community, work or policy environments, or in a combination of settings [7].

It is known that the views of family and social networks, and community attitudes and beliefs particularly around breastfeeding in public, can influence parenting and infant feeding decisions [8]. This appears to be particularly so in disadvantaged groups where the difference in breastfeeding initiation and duration rates, compared to those of the most advantaged groups, is widening, potentially compounding the disadvantage for these infants [9]. Women with lower levels of education, who are younger, single, on low incomes or who live in disadvantaged communities tend to start, but not continue to breastfeed [10].

Significant efforts have been made by governments at a global and national level to promote and support breastfeeding and to increase rates of exclusive breastfeeding to six months. However, initiatives such as the Baby Friendly Health Initiative (BFHI) have been difficult to implement across health systems [11] and the emphasis on education of health professionals have not always resulted in a significant increase in the duration of breastfeeding to six months or longer [12]. This may be because many of these initiatives have not been developed from the ‘ground up’ in consultation with women, families and communities.

The BFHI is an evidence-based intervention that is associated with increases in breastfeeding initiation and continuation. However, alone it cannot achieve exclusive breastfeeding for six months [12]. Community-based interventions such as professional support at home or in health facilities, home visits by trained professionals, home based peer counselling, and the involvement of fathers is required [3]. UNICEF UK has prepared the 7-point plan for Sustaining Breastfeeding in the Community articulating the role of non-hospital health facilities in supporting breastfeeding [13]. Various countries have adapted the community policy, for example in Italy, the Baby Friendly Community Initiative (BFCI) includes a role for day care centres and pharmacies [12]. The recent Lancet series on breastfeeding [3] emphasises the societal responsibility for breastfeeding, reinforcing a breastfeeding culture and overcoming restrictions on breastfeeding in public.

In this project we aim to shift the current focus on breastfeeding as primarily a medical or health issue for which individual women and health systems are responsible, to one that is a social concern where communities, including employers have a role to play. The overall goal of the study presented in this paper is to inform the design of community-based principles and strategies to support new mothers to breastfeed their infants and to enhance the early parenting experience. We are interested not only in the role of communities in supporting breastfeeding and parenting, but also in how public spaces are designed and used to facilitate breastfeeding and parenting young children.

Research on the impact of place (neighbourhoods and communities) on health [14] has increased exponentially over the past 20 years and a strong association has been found between neighbourhood characteristics and parent and child health outcomes [15, 16]. Andrews, a health geographer, argues that public places are conceptualised as more than physical locations or boundaries for human activity, and are instead understood as complex social phenomena [17]. Public spaces tend to bring people together, and are a place where friendships and support networks are formed and maintained [14, 18]. In contrast however, attitudes, social norms and cultural opinion about breastfeeding in public spaces draw ongoing attention and controversy. Boyer [19], for example, describes the loss of comfort that occurs when others in public spaces are made uncomfortable by a behaviour (breastfeeding) that is seen as different to their own. Henderson’s [20] study of men living in deprived communities in England supports this. While men perceived breastfeeding as ‘natural’ it was problematic in public spaces, whereas formula feeding was considered convenient and safe. An Australian study by McIntyre and colleagues [21] demonstrated a widespread social disapproval of public breastfeeding; 82% agreed that bottle feeding is more acceptable in public than breastfeeding, although more recently, Meng and colleagues [22] found 70% of respondents to the Infant Feeding surveys in Western Australia said that breastfeeding in public was acceptable. Feminist human geographers however demonstrate the work women must engage in to manage the emotions of others in order to breastfeed their baby in a public space [19].

In this study we take a participatory approach to discover what is working well to support breastfeeding and parenting of young infants in Australian communities. We started this study by working collaboratively with diverse community members and health care consumers in two Local Government Areas (LGA), one in New South Wales (NSW) and one in Victoria, to describe the key components or characteristics of local communities that promote and support breastfeeding and early parenting. In this paper we report on phase one, the discovery phase of this Appreciative Inquiry (AI) project.

## Methods

A participatory methodology known as Appreciative Inquiry (AI) was used to facilitate ‘Community Conversations’ in two local council areas in two different states in Australia, one in NSW and the other in Victoria. This included one workshop in each site and an additional focus group discussion with retail owners and managers from one shopping centre in NSW.

Appreciative Inquiry is a transformative approach to change which focuses on collaboration and identifying and working with the positive aspects of organisations, or communities, rather than the problems [23]. AI was originally conceived by Cooperrider and Srivasta in 1987 and adopts a social constructionist view based on affirmation, appreciation and positive dialog [24]. AI has the potential to be transformational, shifting the focus from problems to be solved, for example, limited community support for breastfeeding, to discovering and building on what works well within an organisation or in this case a community, and using that as the beginning point for change [25, 26]. As a participatory approach, AI offers a framework to facilitate change from the grass roots up [27]. It does this by emphasising the importance of forming effective partnerships and collaborations that can be used to meet particular needs of an organisation or community. The power of positive dialog is emphasized in AI, suggesting that such dialog has the ability to positively influence organisational growth [28]. Generating collective vision and action are considered essential components in bringing about change when using the AI process. AI has been used in various settings including businesses, education, non-government organisations, communities, and diverse health care settings [25, 26, 29, 30]. The authors have previously used AI in a community setting with young parents [31] and in facilitating family-centred care in neonatal nurseries [32].

AI consists of four iterative phases – The 4D cycle – discovery, dream, design and destiny [33, 34]. At the core of the 4D cycle is an affirmative topic choice, which is considered a significant component of the AI process, highlighting that change is implicit in the very first question asked [33]. The discovery phase seeks to explore ‘what gives life’ to individuals, their work and the organisation, through appreciation and valuing what is best of what is or has been [33, 34]. The dream phase focuses on envisioning ‘what might be’ or affirmative exploration. The dream phase often seeks to elicit insights into individuals and practice through the generation of affirmative stories, usually focusing on recalling peak experiences or high points [33, 34]. Typically, participants are encouraged to visualise how things might look if a miracle occurred, or if they had a magic wand. The design phase focuses on working together to construct the ideal of ‘what should be’ [33, 34] and finally, the destiny phase considers how to sustain what will be or the envisioned future [33, 34]. This paper reports on the outcomes of the discovery phase of this study.

### Study sites

Both study sites were located in outer suburban areas in either Melbourne or Sydney. Site 1 had a diverse multicultural population whereas in site 2, while many migrant and refugee families were moving to the area, the community was predominantly Anglo-Australian. Both sites had one of the lowest breastfeeding initiation rates in their respective States.

### Participants and recruitment

We aimed to recruit 20 to 25 community members to participate in the three-hour long workshops. It was envisaged that participants would include new parents, grandparents, peer support groups such as Australian Breastfeeding Association (ABA), children’s services (playgroups, preschools), local government, local business representatives such as retail managers, café owners or staff, members of church groups and representatives from local maternity and child health services.

Staff and project partners from the two local councils recruited workshop participants by sending out letters of invitation via email or post or through telephone contact to community groups, services and individuals who are well-networked in the community. To encourage participation, Council staff re-contacted invitees in person, by phone or email to remind them about the workshop. We experienced some difficulty recruiting local business owners or managers and staff to the workshops with only one business representative attending at site 1 (conducted in the evening). However as described below, participants in both workshops discussed the importance of the shopping centres as a meeting place for parents and that these sites were particularly important for supporting breastfeeding. We therefore decided to conduct a focus group with retail managers and workers. This focus group was only offered in site 2 as the research team were not in a position to travel to Victoria again. Retail participants at site 2 were recruited by the Centre Communications Manager.

### Data collection

Data were collected via two community conversations held in workshop format and one focus group conversation with retailers.

#### Workshops/community conversations

Both the workshops were held in a community centre in the respective LGA. One workshop was held in the day commencing at 10 am until 1 pm and lunch was served. The other workshop was held in the evening from 6 pm to 9 pm starting with a buffet dinner at 6 pm. The room was set up with four tables each intended to seat five to seven people with space to move around for small group activities.

The workshops were facilitated by experienced facilitators. At site 1, a group facilitator was employed externally to the research group and at site 2, a research team member facilitated the larger group with support from other team members. In each workshop the participants were seated around tables in groups of four to five participants with one facilitator. The 16 participants in site 1 were divided into three small groups and the 19 participants at site 2 were placed in four small groups. Questions were developed by the project steering committee and were in line with the phases of the AI process. The first phase of the forum (AI discovery phase) focused on questions eliciting what is currently working well to support new families and breastfeeding, for example, “What is it about this community that makes it really great for parents with young babies?”. This prompt about parenting an infant in general was followed by prompts related to breastfeeding, for example, “What is it about this community that makes it possible for women to breastfeed in public spaces?” In the second AI phase (the dream phase) participants explored collectively their hopes and dreams for a community that can support mothers to breastfeed. In the third AI phase (design) participants were asked to identify strategies that will assist the community to achieve the vision and principles articulated in the dream phase and to plan for the short, medium and long term future (AI destiny phase). This paper reports on Phase 1 (the discovery phase). The small group discussions at each table were audio-recorded and the brainstorming sessions and feedback were documented in fieldnotes. The audio-recordings were not transcribed verbatim for two reasons; first the participants were generally comprehensive in what they documented on the butcher’s paper and post-it notes and in the feedback to the larger group, which was recorded in fieldnotes. Second, the quality of recording was variable as there was a lot of noise in the room from the workshop groups.

#### Focus group

We had the opportunity to conduct a separate focus group with retail managers from the large shopping centre in site 2. This was organised for two reasons, first no business or retail representatives were available to attend the workshop at site 2 and second, the local shopping centre featured strongly in the two community conversations and it was important to gain retailers’ perspectives. The group was held in a meeting room in the management area of the shopping centre. The same key prompts used in the AI workshop were used to stimulate discussion. However, the focus group differed from the two workshops in that we did not conduct separate small group activities and the time was restricted to one hour. The focus group was audio-recorded and transcribed verbatim.

### Data analysis

All qualitative (textual) data from the community conversations and related fieldnotes were transcribed (where audio recorded). Qualitative content analysis [35–37] was selected as the most appropriate way to code and analyse the descriptive textual data recorded in the workshops on butcher’s paper and post-it notes during the group activities and fieldnotes from the feedback sessions. Focus group transcripts were analysed in the same way and the datasets were combined. Hsieh and Shannon define qualitative content analysis as “a research method for the subjective interpretation of the content of text data through the systematic classification process of coding and identifying themes or patterns” ([37] p 1278). Four stages have been identified in this approach to data analysis – *decontextualisation, recontextualisation, categorisation,* and *compilation* [35]*.* Informed by Hsieth and Shannon [37] and Bengtsson [35], we conducted a conventional qualitative content analysis where we first observed what was happening in the data by reading and rereading the data recorded on the butcher’s papers, post-it notes and the fieldnotes taken during the feedback discussion in the discovery phase of the workshops and the focus group. We also listened to the workshop recordings to see if there were any additional ideas, experiences or concepts that participants did not report in the feedback session. During this process we also identified preliminary codes and labelled individual pieces of data. Similar codes were then grouped together, recontextualised and then categorised into the emerging themes. The theme of PLACE was manifest throughout the data and the developed codes and thus became the central core of each theme.

## Results

In total 35 individuals participated in the two workshops. Participants included eight parents of young children, three of whom brought their children with them to the day time workshop at site 2. One father who was the partner of a participant with a young child also attended at site 1. Three of the attending parents were members of the ABA; two business representatives (one from a local bank and one from a food outlet); representatives from diverse community services including services for migrants attended as well as health professionals including two midwives and four child and family health nurses; managers of the key council services were also present. Staff from the local councils also attended. In addition, six retailers participated in a focus group. These retailers comprised four women and two men and they represented cafes, restaurants, women’s section in a department store. The Community Relations Manager from the shopping precinct also participated (see Table 1).

**Table 1:** Workshop and focus group participants

Site 1 (total 16)	
Participants (unless otherwise stated were women)	N
Parents of young children (3 mothers, 1 father)	4
Local Council – child health staff	3
Local Council – Children’s Services Manager	1
Local Council – Planning and Design Manager	1
Community representative and grandfather	1
Non-Government Organisations – supporting culturally diverse and vulnerable families	4
Local Bank Manager	1
Café manager	1
Site 2 (total 19)	
Participants (all women)	**N**
Parents of young children (4 mothers, 5 children)	4
Health Service – Hospital Maternity Service	2
Health Service – Community Child and Family Health	3
Health Service – Community Allied Health and Aboriginal health	2
Local Council – Children’s Services and Library Services	3
Local Council Planning	1
Non-Government Organisations supporting families	3
ABA representative	1
Focus Group Site 2 with Retail Managers/Owners (total 7)	
Participants (unless otherwise stated were women)	**N**
Shopping precinct Community Relations Manager	1
Cafe manager	1
Restaurant manager (male)	1
Bookshop manager (male)	1
Bookshop and toyshop	1
Maternity and children’s wear outlet	1
Department store manager	1

**Fig. 1:**
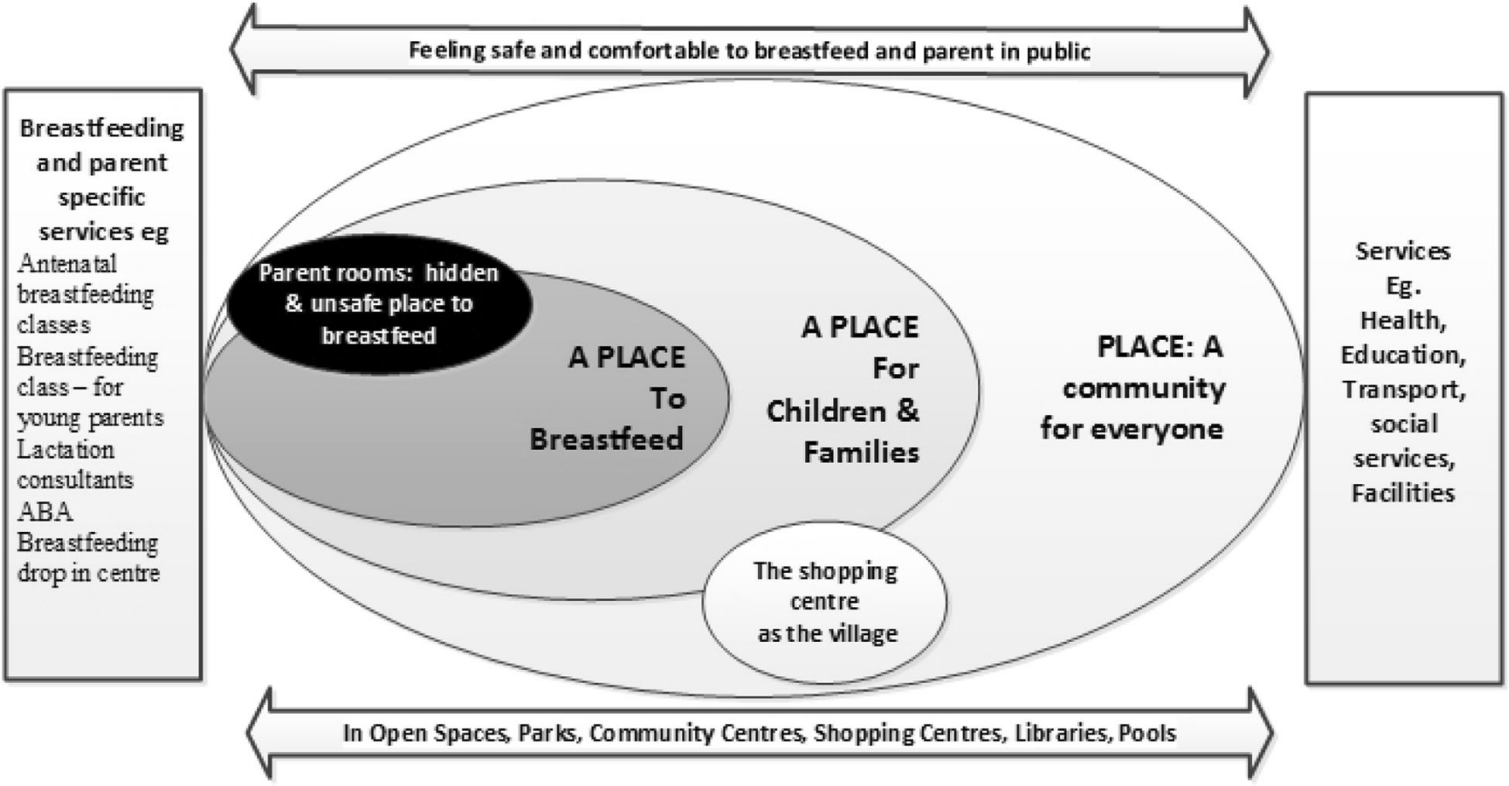
Place of sanctuary: communities supporting breastfeeding and parenting in public

The concept of PLACE and being in certain places in their communities as a mother with a baby or young children permeated the conversations and the focus group discussion with retail managers and owners. Four themes emerged from analysis of the workshop and focus group data that represent a continuum of inclusive and comfortable places to places that were hidden and unsafe: “PLACE – A community for everyone”; “A PLACE for children and families”; “Sometimes a PLACE to breastfeed” and “The parent room: a hidden and unsafe PLACE to breastfeed”. The analysis is illustrated in Fig. 1.

### PLACE – a community for everyone

Participants commenced the conversations by speaking broadly about the positive characteristics of their communities and the services that fostered health and well-being for all community members including children and families. For example, in one community conversation participants stated, “*Our community embraces people and provides all the services that community members need*” (site 1 group 2). Both groups highlighted the work that people within their community undertake to support and develop their communities. Participants in site 2 noted “*there are active community groups that work hard to make our community a great place*” (group 1) and in site 1, participants believed theirs was “*a community where people volunteer to help their fellow community members*” (group 3).

Of particular importance in conversations at both sites was access to green spaces, “*we love that there are lots of parks, walking tracks, open spaces*” (site 2 field note). Being on the peri-urban fringe one group of participants indicated that access to semi-rural areas provided “*opportunities for children to see farms and farm animals*” (site 2 group 1). Green space was also important in the shopping precincts and one retailer manager commented, “*The little park area outside the food court is always nice. You see a lot of families that have gathered there just to get a bit of sun, but this space could be enhanced with more connection to the centre*” (site 2 focus group).

There was also mention of the positive efforts that local businesses and government can make to accommodate or reflect community needs. For example, in site 1, participants noted that the banks will “*hire staff to match community – in the bank they have so many languages spoken, they really match the community needs because the bank staff can speak 14 different languages*” (group 3).

The role of local government in bringing communities together was emphasised and highly valued. The local council was described as “*facilitating opportunities for community members particularly new arrivals to share experiences*” (site 1 group 1). Councils also provided services and activities that not only build skills but help people to network, “*This local government provides services for all community members, for example workshops for cooking and urban sustainability, libraries and public pools, literacy classes*” (site 2 group 2). They also provide access to computers in community places. It was noted that sometimes “*it is difficult to get the information about these events*” (site 2 fieldnotes).

In site 1 there was discussion of service hubs, where people could go to one place to access a range of services, “*you can access all you need in one place, centralised services including Centrelink, medical centres, the train station, leisure activities, schools, technical and further education, computing facilities and a youth centre”* (group 2). In site 2 participants noted the ease of “*access to transport, to health services, to education including a university campus*” (group 3). This sense of community and service infrastructure formed the basis for a place or community that welcomed children and families and with the potential to facilitate breastfeeding.

### A PLACE for children and families

Participants reported that both communities, particularly the local government services, recognised the importance of the early years for child development and both sites reported that the local council had a plan for fostering health and development in the early years.

Table 2 provides a summary of the core components, activities or services that supported families and could or did promote breastfeeding. These data were provided by participants in the small groups in the two workshops, in fieldnotes recorded during feedback sessions and in the focus group with retail managers and owners. These are organised under the following categories: “Community facilities for children and families”; “The shopping centre as the village”; “Services for diverse community groups”; and “Health services”.

**Table 2:** Core components, activities or services that support breastfeeding and early parenting

Community facilities for children and families*	The shopping centre as the village**	Services for diverse community groups*	Health services*
• Green spaces, walking tracks • Playgrounds with well-maintained, safe equipment • Community centres • Community notice board • Access to parking for parents • Mothers groups • Australian Breastfeeding Association • Playgroups collocated with services • Local council events • Access to child care • Information for parents – mailed to each parent in community, annual parent directory, new resident welcome packs • Federal government local member welcome to new babies event.	• Cafes and restaurants welcoming and never being difficult in terms of supporting breastfeeding • accepting of children • shopping centre family room • play areas • open spaces for kids • ‘POP UP’ playgroups in shopping centres • Places available to feed babies • Lots of facilities have baby change areas	• Specific services for Aboriginal women • Community groups e.g. Turkish women’s groups • Young parent program for example a specific mentoring program • Culturally specific playgroups • Bicultural workers • Interpreters available and free to families	• World class maternity hospital • Antenatal classes • Breastfeeding class for young parents • Caseload midwifery • Midwifery @ Home • Handover from hospital to community services • Mentoring program – MASE • Child and family health nursing services are available for parents and these nurses were considered knowledgeable and worked flexibly in the community • CFHN helpline • Breastfeeding drop in centre • Immunisation clinics in community settings • Good GP services Other services: • ABA helpline
	Key *Data generated at the workshops at sites 1 and 2 **Data generated through the focus group with retailers as well as through the workshops	Other community services*	
		• NGO and others services meeting and collaborate • School hubs – support for whole family; provide adult education including language; offer employment pathways	

#### Community facilities for children and families

In workshop groups that had more parent, local government and business participants rather than health and community service providers, the discussions focused mostly on the local environment and facilities for children and families including easy access for parking or for transport. These participants painted a picture of their community as a healthy place to bring up children. As noted above, they valued access to parks, walking tracks and open spaces as well as community facilities such as playgrounds with well-maintained, safe equipment for families with young children. Community centres or hubs that are safe and comfortable for parents were important. One father and one grandfather in attendance also noted that the “*community ensured they were valued as fathers by providing access to parenting rooms and offering some father specific services or events*” (site 1 group 1).

Participants highlighted the value of the children’s events saying: “*diverse children’s events are run by the Council as well as individual groups who run things in other locations such as the shopping centres*” (site 2 group 4). In both sites they described the *Munch and Move program, Paint the town Read* and book week (sites 1 & 2 fieldnotes).

Participants in site 2 described the role of the local council in:

*...linking mums with peers and there are playgroups, including a mobile play van from council and this service puts out chairs for mothers so there is somewhere to sit when feeding. The ABA is very active in this area and some local churches run groups* (group 2).

Local Federal and State government members of parliament also got involved. Recently, a Federal local member had held a “*Welcoming babies*” event (site 2 fieldnotes).

It was important that information was available to parents using diverse mediums for example in one site there was a “New Parents” page in the local newspaper (site 1 fieldnotes) and a participant at site 2 stated: “*currently there is a regular community newsletter that highlights topics about child health and parenting such as eye checks, play therapy, podiatry, community centres, and information about services*” (group 2). There is a local annual Parents’ directory that was in hard copy, although participants noted the “*difficulties of keeping that updated and found that online resource directories were more important*” (site 2 fieldnotes). Brochures were available in childcare centres with information on a range of topics. Council run children’s events and the library were also great places for parents with young children and the local pools were seen as family friendly.

Gaps and challenges were also identified. For example, there was mention that *“parking in some areas, where community based programs are run is very limited”* (Site 2, group 1)*,* and that “*playgroups were not available in all areas”* (Site 2 group 3). The following participant described that “*currently there is no playgroups and nothing for parents in walking distance in XXX suburb itself they are all out here*” (site 2 group 1).

#### The shopping Centre as the ‘village’

In site 1 the local shopping centre was described as having a ‘village’ atmosphere, something that had developed over the past few years, and this was reflected in the recent offering of ‘pop up’ playgroups in the shopping area. Participants indicated that there were “*nice places to sit with comfortable chairs*” (site 1 group 1). In both sites the shopping centres were seen as places that you could go and meet up with friends, have a coffee, find something for the children to do and to keep warm in winter and cool in summer (sites 1 & 2 fieldnotes).

Participants also reported however that access for parents in shopping centres in general could be a problem. For example, it was difficult to navigate the shopping centre with prams:

*You quite often see people struggling to get through. People are coming out of the bathrooms and coming out of the eatery as well. It’s a little bit hard* (site 1 group 3).

*The elevators are small. It’s the smallest elevator in the area. You put two strollers in there and the elevator’s full and our parents’ room and our playground is upstairs. They have to use the elevator* (site 2 group 1).

Parents also reported it was difficult to access spaces when they had to move through heavy doors with prams, for example in site 2 group 1, the following conversation was recorded:

Participant 1: *To go into the doors are so heavy! Can’t open with pram and kids.*

Participant 2: *You end up banging into everything? How are you going to open the doors? You’re pushing them open.*

Places such as the parent rooms could also become busy and crowded:

*It gets very cramped. There are really good mother’s rooms down this end of the centre that are quite good. They’ve got microwaves and everything else in there but it can get quite packed down with people trying to shuffle in and out of that corridor* (site 2 fieldnotes).

Because of the limited facilities some parents indicated that they preferred other shopping centre facilities:

*I have to say our local area doesn’t do it as well as some other areas. I shop at XXX and it’s like kids behind glass gates. You sit in your big lounge chair breastfeeding and you look through the glass gates and there’s soft play equipment and TVs*. *.*. *Shall we say here in the shopping centre it is minimal*. *.*. *Basic* (site 2 group 1).

Sometimes the shopping centre was not seen as parent friendly and one retailer described:

*I see more negative that side. I normally see people looking down their nose or commenting on other people. That whole do you get involved, get involved thing. If a child’s loud, annoys you, or playing up or whatever, you tend to see a lot of disapproving looks around the place. I see more disapproving than sympathy, from what I’ve seen* (site 2 focus group).

#### Breastfeeding and parenting specific services

The conversation differed slightly in the group that comprised more health service professionals (site 2). Rather than focusing on the community, facilities and resources in a community, these participants (group 4) were more likely to list the diverse range of health services available to pregnant women and parents with new babies and young children (see Table 1 column 4). In both sites, it was identified that antenatal classes are available through the hospital and these include information about breastfeeding and both sites offered either a community-based or hospital-based breastfeeding session for young parents. Other health services included outreach and residential parenting services, General Practice shared care for maternity care and there were also specific services for culturally and linguistically diverse families and families who have additional needs.

Services for children and families in site 1 were described as being culturally aware and appropriate for young families. The child and family health nursing service was described as “*an excellent ongoing resource for families and the nurses were viewed as knowledgeable and encouraging*” (site 1 fieldnotes). The importance of access to bicultural workers was also mentioned as well as the Young Parent Program.

However, despite the very positive perceptions of participants about their communities, they did not believe that breastfeeding was well supported and they were particularly critical of facilities in shopping centres. The theme “Sometimes a PLACE to breastfeed” captured this negative aspect of the community place narrative.

### Sometimes a PLACE to breastfeed

Participants in both sites lamented that the breastfeeding rates in both communities were lower than they should be. The statements below capture the available support for breastfeeding in these two communities. Overall support was deemed limited, with the provision of support seen as a case of only “some” or “sometimes”:

- **Some** cafes restaurants welcome breastfeeding mothers (sites 1 & 2 fieldnotes; focus group)
- **Some** shops have the Breastfeeding Welcome Here stickers (site 2 fieldnotes; focus group)
- **Some** parent room facilities are comfortable for feeding (site 2 fieldnotes)
- There are **some** identified breastfeeding friendly workplaces in the community (sites 1 & 2 fieldnotes, focus group)

Participants reported that breastfeeding is rarely observed in public, “*bottle feeding is far more common, you just don’t see breastfeeding*” (site 2 fieldnotes) and at site 1 participants acknowledged: “*it might be that many of the women from diverse cultural backgrounds are not comfortable feeding in public but you just don’t see it*” (group 2). Participants believed that modesty and body image influenced local mothers’ decision to breastfeed in public. Early return to work was also identified as a factor influencing low breastfeeding rates.

An alternative view was offered in the retailers’ focus group. One participant who worked in a department store in site 2 stated:

*I don’t find it unusual to see a mum with a small child wandering through the store while feeding. Certainly, it doesn’t raise any issues with other customers. It seems to be very commonplace, at least throughout the children’s department for a mother feeding and a family to be wandering through and having that level of comfort, which I think is certainly a positive. Having worked in a number of centres with and see nothing, the comfort level here seems to be a little more than some of the other centres that I’ve been in, which I think is very positive.*

Participants indicated that they believe the support for women to breastfeed in shopping centres and in other public places was ad hoc and not coordinated. Basic parent rooms and facilities to breastfeed in private were available at most shopping precincts and as indicated above, there were some breastfeeding friendly cafes and sometimes mothers were seen breastfeeding at child care centres or expressing breast milk for children in childcare (sites 1 & 2 fieldnotes).

Concern was also expressed that fathers could not easily access and did not feel comfortable in public places such as parenting rooms, particularly if women were breastfeeding there. On the other hand, some participants stated that men should not have access to the parenting room spaces (sites 1 & 2 fieldnotes).

Importantly the role that ABA played in promoting breastfeeding in the communities was noted in both workshops as well as in the retailers’ focus group, for example:

*Talking to the ladies from ABA, they’ve made it really clear that they’re doing a lot of education work with new and expected mothers about comfort levels and how to make yourself more comfortable. They’ve actually now got a register on their website where businesses can actually be approved. We’ve got a sticker which says we’re breastfeeding approved, which means they’ve had interactions with us and they’re making it clear that mothers can feel really safe breastfeeding in our restaurant because we actually support it and we’ve gone out of our way to support that* (site 2 focus group).

The “Breastfeeding Welcome Here” sticker was observed in the shopping centres. However, participants indicated that *“this support is limited and large numbers of women in this community do not breastfeed their babies”* (site 1). The active presence of ABA appeared to be important in promoting community support for breastfeeding.

### The parent room: a hidden and unsafe PLACE to breastfeed

Places for mothers to breastfeed were hidden away. Parents talked about having to walk down long dark corridors to the parent room facilities and parents in the site 1 workshop commented that they “*don’t like to be tucked away and so they breastfeed quickly*” (group 1).

Privacy and safety were also a concern in parenting rooms. Some mothers expressed concern about men having access to parenting rooms if they are breastfeeding. This was also an issue raised by the retail staff:

*I think entrances that are a little more public, with a degree of privacy, are a lot more appealing because you don’t feel you have to go down a long, winding corner and around a corner where you don’t feel like there’s a lot of people. If you turn around behind you and you see five men walking towards the bathroom you’re probably not going to feel comfortable whereas if you turn around and there’s twenty or thirty people walking past, families, and someone of every age, I think it’s a little more comforting when you walk into it* (site 2 focus group).

On the other hand, participants noted that it can be difficult for fathers to access the parenting rooms if they need to change their child’s nappy or take them to the toilet:

*You know, the other thing I’ve always had trouble with is they will always put the baby change tables in the mother’s toilets. I work full time, my husband cares for our children*. *.*. *where does he change a baby?* (site 1 group 1).

Some participants were worried about security, “*We’ve got security, CCTV cameras in the hallways, we don’t want to put them in the bathroom*” (site 2 focus group). The following conversation in the workshop at site 2 demonstrates discomfort about current parenting room facilities particularly in relation to safety:

Participant 1: *It is difficult breastfeeding when you are out and about and the toilets are not pleasant and at times feel unsafe.*

Participant 2: *. .*. *they are not the best place to go to because they stink and the toilets don’t work. It’s quite frustrating actually*. *. . They’re either broken, or when you go there’s other people watching. There’s people smoking in there or junkies whatever or people without any children.*

Participant 3: *. . . and there is syringes all around. The amount of times I go to the centre and I end up making a complaint and am told “Well there’s nothing that we can do for you”* (site 2 group 1).

Some parents believed they had to make do with what was there and one person added: “*Even basic parenting facilities is better than none*” (site 2 group 2). Others stressed it was important to address some of the issues related to facilities and services in the community because “*It’s about reducing stress so you can enjoy your children*” (site 2 group 2). However, all participants aspired to being in a place where “*breastfeeding is seen as normal and where women are comfortable to breastfeed anywhere and don’t feel the need to have to cover up*” (site 1 fieldnotes).

## Discussion

This study has examined the perceptions of diverse community members including six retail owners or managers about the role of communities in supporting breastfeeding and early parenting. The data presented were gathered in the discovery phase of this AI study with a view to informing the development of principles and strategies of Mother Infant Caring Communities that support breastfeeding and early parenting. ‘Place’ emerged as the central theme in our analysis of the discovery phase. Guided by an appreciative approach, participants first described their community as being a “*place for everyone*” as well as “*a place for children and families*”, where diversity is welcomed and people of all ages from all groups have a way to connect to place. Significant features included available open green spaces, playgrounds, community centres, libraries, diverse community events and activities as well as supporting equal access to quality health, education, employment, transport and social services particularly for vulnerable groups in the community such as elderly residents and new migrants. However, in both sites, breastfeeding was rarely seen in public. Some cafes, restaurants, and public spaces were breastfeeding friendly, facilitating comfort for public breastfeeding but in general, parenting rooms in shopping centres did not meet the needs of breastfeeding women.

The findings from this study support the idea noted in the introduction to the paper that public spaces can bring people together, and support development and maintenance of friendships and support networks [14, 18]. These social interactions are a key element in one’s general sense of well-being. Both meaningful and fleeting interaction can provide relief from daily routines, enhancement for peoples’ sense of community, and alleviate tensions [18]. As demonstrated through these community conversations, social relationships and interactions take place in minor or everyday settings such as at the park, childcare centres, libraries, and recreation facilities, where residents can congregate both informally and formally and observe each other in public [38].

While participants in this study were able to identify the types or characteristics of public places that offer sanctuary for parents of young children in general, and for breastfeeding mothers specifically, other research highlights ways in which public places are experienced as a point of surveillance [14, 39, 40]. The discomfort experienced by many women breastfeeding in public, and the sense of being surveilled or monitored as a good or bad mother, is well documented [41–44]. Bell states that sanctuary is often sought out by individuals who experience a sense of liminality, the feeling of not belonging [14]. Bell draws on the example of people with mental illness or chronic illness, however Mahon-Daly and Andrews and others have identified breastfeeding itself, as well as breastfeeding in public as a liminal experience, feeling “betwixt and between”, out of place or not belonging [45–47]. These authors state that there are places which are acceptable and unacceptable and unacceptable to breastfeed. To find sanctuary and avoid surveillance, women actively seek particular spaces and times to breastfeed [47, 48]. In this study, a sanctuary for parents of young babies would be a place where *“breastfeeding is seen as normal*. *.*.”

### Places of sanctuary

We found that open green spaces, aspects of shopping centres that created a village atmosphere and specific breastfeeding services, or spaces, offered breastfeeding women and parents sanctuary when they were in public. On the other hand, spaces such as parent rooms and other public places were often unsafe and potential sites of surveillance.

### Green spaces

Open green spaces, parks and walking tracks were a central feature in both communities and benefitted everyone in the community. Features such as safe playgrounds where children could be observed while a mother was breastfeeding and walking tracks with seats offering quiet places for breastfeeding were valued. This is supported by a recent study comparing mothers’ experiences of raising young children in inner and outer suburbs in Melbourne that highlighted the importance of parks as a shared space where mothers could connect with other parents [49]. Cattell et al. described the important role of parks for well-being, considering them to be places of escape, to unwind, participate in leisure activities, observe others, seek solitude, or to simply go for a walk [18]. For a place to be perceived as beneficial to one’s well-being it needs to have several basic properties: security, provide a feeling of identity, material wants, and aesthetic pleasure. In this way people can build trust in their community [18] and feel a sense of belonging. Contact with green spaces is also associated with mental health benefits. Studies show an inverse relationship between the amount of contact with green space and levels of stress [14, 50]. A recent systematic review suggests that contact with nature, in green spaces, can also have a positive effect on blood pressure, heart rate, skin conductance and muscle tension [50, 51]. The study by Roe and colleagues found that women living in neighbourhoods with lower levels of green spaces experienced significantly higher levels of perceived stress than women living in areas with a high level of green spaces [51].

### The village

The shopping centre, whether a large complex or a smaller local centre, was a focal point for families with young children. Participants used the term, ‘the village’ to describe these places, highlighting both the social and pragmatic function of shopping centres. The positive features of shopping centres included access to breastfeeding and family friendly cafes, restaurants and shops. The level of comfort that some parents experienced in these centres was demonstrated by the capacity of groups of mothers to create their own space, for example in cafes, where they could move chairs and tables around to create a more intimate space. Boyer [39] similarly noted that coffee shops with couches, movable comfy chairs and dim lights are an enabling factor in breastfeeding outside the home. These positive aspects were also noted and endorsed by the retailer managers and owners who participated in the study.

There is little research on the role that shopping centres play in the daily life of parents of young children and particularly about how breastfeeding is supported in this context. The most common accounts report negative experiences where women have been asked to cover–up or to go elsewhere to breastfeed their baby [52]. A recent study [53] investigated the child friendliness of 62 urban shopping centres in Poland and found parents with children under three years prioritised well equipped and clean baby changing and feeding rooms which are separated from each other (as mothers do not want to feed their children in toilets), toilets adapted for children, vending machines with nappies, as well as parking spaces for families with prams. They also appreciated access to first aid supplies and centres that consider safety issues.

Only six self-selected retail owners or managers participated in the study and all expressed positive views towards breastfeeding. They described what they thought was currently working well, for example, the display of “Breastfeeding Welcome Here” stickers as well as positive interactions between some food outlets and the ABA. They also recognised there was room for improvement, particularly in the parenting room facilities. There is limited research examining retailers’ perceptions of how to support breastfeeding in their outlets. In the late 1990s, McIntyre and colleagues conducted a survey in Australia of restaurant and shopping centre managers concerning breastfeeding in their facilities [54]. Ninety-three restaurant and shopping centre managers completed a structured telephone survey. One-third of the restaurant managers and 48% of the shopping centre managers stated that a mother could breastfeed anywhere in their facility regardless of what other customers might say [54]. The remaining managers would either discourage breastfeeding anywhere in their facility, suggest a mother move to a more secluded area if she wished to breastfeed, or were unsure how they would react [54]. More recently in the UK, Marsden and Abayomi examined a small group of employees’ attitudes and opinions toward women breastfeeding in public [55]. All were employees of a shop, restaurant or cafe and job roles varied from managers to general team members. Most participants expressed positive views about breastfeeding, particularly those who had experience of breastfeeding themselves. They indicated they would protect the rights of women who wished to breastfeed in their establishments should they be challenged by members of the public. However, across two decades these studies demonstrate that the pressure remains with women to manage breastfeeding in public in a discrete and acceptable way, and the perception that breastfeeding women feel uncomfortable breastfeeding in public and require private facilities for breastfeeding to alleviate their anxieties remains [55].

### Special breastfeeding places

While open green spaces, safe playgrounds, community centres and shopping centres may offer a sanctuary for parents with young children and sometimes for breastfeeding, participants were most appreciative of places, services and programs catering specifically for breastfeeding mothers. These places included mother’s groups (health-led or ABA), breastfeeding drop in centres, culturally specific playgroups, and maternal, child and family health clinics which offered a sense of comfort and belonging, a form of sanctuary away from public view.

In the UK, Children’s Centres have been described by both mothers and staff as places supportive of breastfeeding, making women feel confident, and without fear of being criticized [56]. There is also a long tradition in Australia and elsewhere of facilitated mothers’ groups. Typically, in Australia these groups are offered by local maternal, child and family health nurses [57, 58] as well as the local branches of the ABA [59]. These groups conducted in diverse community settings have been positively evaluated for their impact on mothers’ well-being, connection to community and as places that offer mothers comfort to breastfeed either in the group or in a separate location outside of the group [57, 60–62]. There is also some evidence that attendance at new mothers’ groups support breastfeeding continuation [58].

A breastfeeding drop-in centre was available in one site in this study. These centres, sometimes called baby cafés, are community-based initiatives, however in most instances they are established by health services and facilitated by health professionals similar to a new mother’s group. Evaluations of these services demonstrate that women value the social aspect of the Baby Cafe service and benefit from interactions with other breastfeeding mothers, as well as from specialist expertise to address specific feeding difficulties [63]. As part of a large three arm trial, Cramer and colleagues [64] reported on the outcomes of several breastfeeding drop-in centres facilitated by peer supporters from the ABA and a maternal and child health nurse. They found that in some disadvantaged locations there were challenges in attracting mothers to use the breastfeeding drop in centres and some centres had not continued following the trial. Fox reported that services such as the Baby Cafe tend to attract older, more advantaged mothers and those with a strong initial commitment to breastfeeding [63]. It can take some time for these support centres to become established in a community. Cramer [64] reported on a range of complex factors that impacted on the establishment of the drop-in centres and also the attendance by women including accessibility, available space, recruiting volunteers to provide peer support, and frustration when women did not attend.

As noted, these services and supports described by participants are typically initiated and facilitated by health staff. Women themselves have also created alternative spaces for breastfeeding. For example, the breastfeeding van in the US that will come to public places when a woman is in need of a place to feed her infant [65]. Women in the UK have also established the mobile phone “app” called Feedfinder [66]. Simpson and colleagues found that an important factor for women included the level of privacy available and qualities of a venue [66].

### Hidden and unsafe places

The purpose of this study was to identify what it was that worked well in communities to support breastfeeding. However, participants also identified what made it difficult or uncomfortable for mothers to breastfeed in public and much criticism was levelled at parenting room facilities. These were places where mothers did not always feel safe or comfortable, and resented being hidden away. While breastfeeding in public is legally supported and generally accepted in Australia, it remains an activity that must be hidden [67, 68]. This view reinforces the responsibility that is placed on breastfeeding mothers to somehow manage others’ discomfort about breastfeeding in public by covering up or preferably removing herself to other locations such as a parenting room [39, 69]. It is unfortunate that because of this dominant discourse and surveillance practices surrounding public breastfeeding [40], that women and their babies must hide in parent rooms that feel unsafe and are often unclean. As Ahmed argues, these affective environments continue to give the message to breastfeeding mothers that they do not belong in a given space [69]. Cook asks whether a space that prioritises the comfort of strangers over the ability to safely breastfeed, is actually ‘public’ at all [40]. She argues that these practices contribute to inequality and a loss of autonomy [40]. Boyer emphasises how the experience of being considered a ‘discomforting other’ contributes to the non-belonging of breastfeeding women in public spaces [39]. Both Cook [40] and Boyer [39] assert that the patriarchal norms that have historically governed norms of public comportment and motherhood continue to influence the coding of public spaces, requiring mothers to divorce themselves from bodily maternal processes, or risk censure.

### Limitations

This was a small study of diverse community members in two LGA. The majority of participants were either active, engaged community members, or had a role in local councils or health services. Three of the eight parents who attended were active members of the ABA and had a level of confidence to participate in this type of workshop. Most therefore had a strong motivation to support breastfeeding. Only four men participated in the study – one father and one grandfather in the workshop at site 1 and two men in the focus group. In further research, it will be important to seek a greater diversity of participants. While the appreciative approach taken in this study is a strength, bringing diverse community members together to ascertain what is working well, in this paper we have only reported on the discovery phase of this project. In ongoing research, our team is conducting additional workshops in other locations and will report on the dream and destiny phases with the aim to describe the principles and strategies of a Mother Infant Caring Community.

### Implications

The study findings contribute to our understanding of how a community and the physical environment supports breastfeeding. The finding that safe green spaces and facilities at shopping centres can facilitate breastfeeding in public together with specific breastfeeding support is important and emphasises the role that urban planning and design have in improving public health. However, as an ecological approach demonstrates, addressing only one element or domain of influence - the community - will not by itself increase breastfeeding duration. Multicomponent and coordinated strategies are required to support breastfeeding. The ‘Breastfeeding Gear’ model is one recent example [2]. This ‘complex adaptive systems’ approach involves strategies that protect, promote and support breastfeeding from pre-pregnancy to birth, the postnatal period and childhood, and in multiple settings and we would argue settings that are beyond the health sector. Effective strategies include policy advocacy and legislation, increasing community awareness regarding breastfeeding, hospital or health-system support through the BFHI approach. We would argue however, that more focus is needed on the role of community mobilisation and workplace support to promote optimal breastfeeding practices. Appreciative Inquiry as a method to engage diverse community members can be helpful to identify what is working to support breastfeeding and parenting and to emphasise and extend these practices, services and facilities. It is also important that the global infant feeding strategies such as BFHI and UNICEF UK 7-point community plan are linked to other initiatives such as Mother Friendly Hospitals and the WHO Child Friendly Cities initiative.

Further collaborative research with health geographers and urban planners is needed to explore how public spaces are used by different cultural and social groups, and the extent to which spaces are shared and may influence community cohesion, and the meanings that parents of young children attach to places.

## Conclusion

This was a small study of diverse community members in two local government areas. The participants articulated what was working well in their communities and where change is needed if parents are to be supported with breastfeeding. Most importantly, the groups articulated a vision that would not only support breastfeeding but would build healthy and happy communities. Further work is needed to identify the strategies that would achieve this ideal.

## Data Availability

The datasets used and/or analysed during the current study are available from the corresponding author on reasonable request.
